# Methods for extracellular vesicle isolation from cancer cells

**DOI:** 10.20517/cdr.2019.118

**Published:** 2020-04-25

**Authors:** Israa Salem, Nicole M. Naranjo, Amrita Singh, Rachel DeRita, Shiv Ram Krishn, Luca S. Sirman, Fabio Quaglia, Alexander Duffy, Nicholas Bowler, Aejaz Sayeed, Lucia R. Languino

**Affiliations:** ^1^Department of Cancer Biology, Sidney Kimmel Cancer Center, Thomas Jefferson University, Philadelphia, PA 19107, USA.; ^2^(Present Address) Astellas Institute for Regenerative Medicine, Marlborough, MA 01752, USA.; ^3^(Present Address) School of Veterinary Medicine, University of Pennsylvania, Philadelphia, PA 19104, USA.; ^4^(Present Address) Baruch S. Blumberg Institute, Doylestown, PA 18902, USA.; ^#^Co-equal first authors.

**Keywords:** Extracellular vesicles, differential ultracentrifugation, iodixanol density gradient, cell culture medium, plasma, tissue

## Abstract

Cells are known to release different types of vesicles such as small extracellular vesicles (sEVs) and large extracellular vesicles (LEVs). sEVs and LEVs play important roles in intercellular communication, pre-metastatic niche formation, and disease progression; both can be detected cell culture media and biological fluids. sEVs and LEVs contain a variety of protein and RNA cargo, and they are believed to impact many biological functions of the recipient cells upon their internalization or binding to cell surface proteins. It has recently been established that standard isolation techniques, such as differential ultracentrifugation, yield a mixed population of EVs. However, density gradient ultracentrifugation has been reported to allow the isolation of sEVs without cellular debris. Here, we describe the most common methods used to isolate sEVs from cell culture medium, mouse and human plasma, and a new technique for isolating sEVs from tissues as well. This article also provides detailed procedures to isolate LEVs.

## 1. Introduction

Cells are able to communicate with each other through the shedding of extracellular vesicles (EVs), which include small extracellular vesicles (sEVs) and large heterogeneous vesicles^[1-4]^. sEVs are secreted *in vivo* by various cell types into biological fluids, including blood, urine, and *in vitro* in cell culture medium^[5]^. sEVs are enriched in proteins such as TSG101, Alix, and tetraspanins (CD9, CD63 and CD81), which are referred to as sEV markers, and they also contain a wide variety of RNAs including mRNA and miRNA^[1,6,7]^. Large extracellular vesicles (LEVs) are found in almost all human biological fluids including cord blood, urine, saliva, amniotic fluid, cerebrospinal fluid, synovial fluid and vitreous fluid^[6]^. Although endoplasmic reticulum proteins are present in LEVs and not in sEVs, there is still a need to find specific markers for LEVs^[8]^. It has been shown that matrix metalloproteinase 2, cytokeratin 18, flotillin-1 and -2, ARF6, and CD40 are markers associated with LEVs^[8-10]^. In this article, the designation LEVs includes large and intermediate-sized EVs as coined by Mathieu *et al.*^[1]^.

sEV development starts in multivesicular bodies, which are formed by the intraluminal inward budding of endosomes. These intracellular vesicles frequently fuse with the plasma membrane of the cell, to be released into the extracellular space. LEVs are formed through budding and fission of the plasma membrane^[2,6]^. The process entails LEVs being pinched off the membrane through the correct arrangement of acto-myosin-driven fission and other components of the cytoskeletal system^[9]^.

The size of EVs, including sEVs and LEVs, ranges from a few nanometers to a few micrometers^[5]^; sEVs are 50 to 200 nm while LEVs may be as small as 200 nm or as large as 1 µm^[2,5-7,11]^. Physiologically and pathologically, sEVs demonstrate their potential through the numerous roles they play in cell-cell communication by transferring bioactive molecules that may lead to disease progression^[8,12]^. They have also been seen to play a role in the regulation of immune functions and inflammation as well, and can participate in organ-specific processes^[13]^. LEVs have also been found to condition the extracellular microenvironment and to promote pathogenesis^[9]^. LEVs are involved in a number of biological functions including, but not limited to, cell adhesion and migration, waste management, vascular function, coagulation, bone calcification, and tissue repair^[3]^.

To utilize EVs in research, it is important to follow the guidelines proposed by the International Society for Extracellular Vesicles reported in the Minimal Information for Studies of Extracellular Vesicles (MISEV 2018) publication. MISEV 2018 prompts researchers to be careful on their analysis of EVs and provides steps and protocols that can be followed to adequately document the biogenesis, uptake and functions of EVs^[5]^. Investigators are also encouraged to use EV-TRACK, which is a knowledgebase of parameters to increase the accuracy of information in the EV-related field. EV-TRACK allows communication between researchers to enhance experimental reproducibility and exchange of knowledge of EVs in general, and it allows queries between researchers on published experiments^[14]^.

Here, we describe the procedures for successfully isolating sEVs from cell culture medium, plasma or tissues using differential ultracentrifugation and iodixanol density gradients. In addition, we illustrate the isolation of LEVs from cell culture medium using differential ultracentrifugation and iodixanol density gradients. Finally, we discuss the isolation of sEVs using an immunocapture technique.

## 2. Isolation of sEVs from cell culture medium via differential ultracentrifugation

### Materials

1. Laminar flow hood.
2. Phosphate-buffered saline 1X (PBS).
3. 0.05% trypsin.
4. Cell culture medium containing *L*-glutamine supplemented with 10% fetal bovine serum (FBS) and 1% Penicillin/Streptomycin (P/S) reagent.
5. FBS-free cell culture medium.
6. Sterile cell culture plates.
7. 15-mL and 50-mL conical tubes.
8. Sterile 10-mL and 25-mL serological pipettes.
9. Tabletop Eppendorf centrifuge with temperature control (5415R).
10. Beckman polycarbonate ultracentrifuge tubes, 70-mL (Cat. # 355620), including cap assemblies, for use with 45Ti rotor.
11. Beckman 45Ti fixed-angle rotor (Cat. # 339160).
12. Weighing balance.
13. Beckman L8-70M Ultracentrifuge (for use with 45Ti rotor).
14. RIPA lysis buffer: 50 mM Tris, pH 7.5, 150 mM NaCl, 1 mM EDTA, pH 8, 1% NP-40, 0.5% Na-deoxycholate, 0.1% sodium dodecyl sulfate (SDS). Add protease inhibitor cocktail just prior to cell lysis: PMSF (1 mM), aprotinin (10 µg/mL), leupeptin (10 µg/mL), pepstatin (4 µg/mL), calpain inhibitor (1µM), and sodium orthovanadate (1 mM).

### Methods

**Figure 1:**
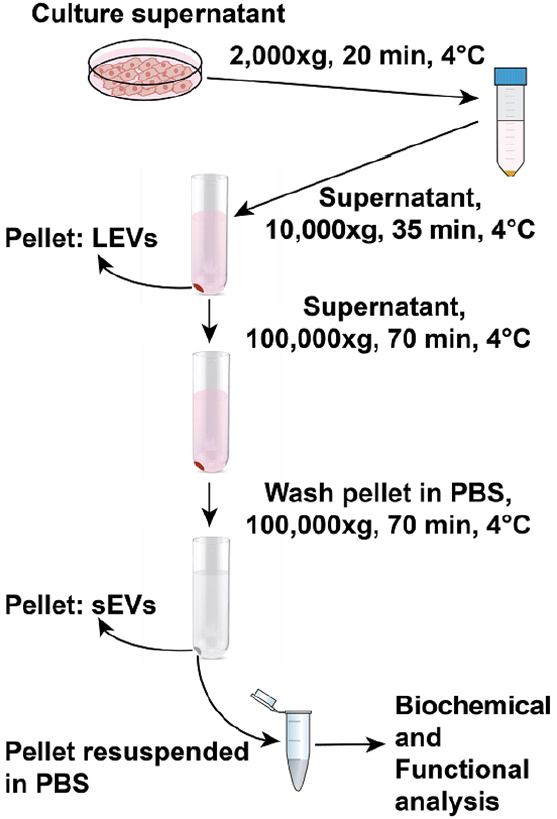
Schematic drawing for extracellular vesicle isolation procedure by differential ultracentrifugation. For extracellular vesicle isolation, the supernatant is collected from cells cultured in dishes for 48 h at 37 °C. In the first step, dead cells and cell debris are spun down from the supernatant at 2000 × *g* for 20 min at 4 °C. The supernatant collected is spun at 9000 rpm (10,000 × *g*) for 35 min at 4 °C. The pellet obtained corresponds to the LEVs and contaminating proteins. The supernatant collected without disturbing the 10,000 × *g* pellet is spun at 100,000 × *g* for 70 min at 4 °C. The pellet corresponding to sEVs and contaminating proteins are washed in PBS followed by a second spin at 100,000 × *g* for 70 min at 4 °C. The final sEV pellet is resuspended in PBS and transferred to Eppendorf tubes. The collected EVs can be biochemically and functionally characterized. sEVs: small extracellular vesicles; LEVs: large extracellular vesicles

This procedure has been adapted from a previously published protocol, and modifications have been added as needed^[15]^; a schematic drawing is shown in Figure 1.

1. Culture cells in sterile 150-mm cell culture plates. Grow cells of interest until they reach 70%-80% confluency.
2. Remove the culture medium and replace it with a similar volume of either FBS-free medium or medium supplemented with sEV-depleted FBS. Use as many plates as necessary to obtain at least 70 mL of conditioned medium.
3. After an additional 48 h, collect the supernatant. Cells that grow rapidly may become overconfluent and start dying after 48 h. In this case, either collect the supernatant after 24 h or use medium supplemented with sEV-depleted FBS.
4. Set tabletop centrifuge chamber temperature at 4 °C.
5. In the laminar flow hood, collect medium in 50-mL conical tubes using a sterile 25-mL serological pipette. Conditioned medium from the same cell line can be combined in tubes.
6. Place 50-mL tubes in the tabletop centrifuge and centrifuge them for 20 min at 2000 × *g* at 4 °C to clear dead cells and cell debris.
7. During this spin, perform total cell lysis using RIPA lysis buffer if desired. Store cell lysates at -20 °C or on ice if quantifying proteins and/or analyzing by SDS-polyacrylamide gel electrophoresis (SDS-PAGE).
8. In the laminar flow hood, transfer the supernatant from 50-mL conical tubes to chilled polycarbonate ultracentrifuge tubes. Each tube can be filled with approximately 65 mL of conditioned medium. You can combine supernatants from the same cell line. Discard supernatant from 50-mL conical tubes (leave 2-3 mL at the bottom to ensure the pellet is not disturbed). Note: for collection of sEVs from cell culture medium, the conditioned medium may be kept at 4 °C for up to 1 week, after the first pre-clearing spin. However, you may observe a measurable decrease in sEV protein yield.
9. Weigh polycarbonate ultracentrifuge tubes on a balance. Equalize ultracentrifuge tube weight with sterile PBS for balance during centrifugation.
10. Ensure that rubber stoppers are all in place (45Ti rotor), mark one side of each ultracentrifuge tube with a waterproof marker, orient the tube in the rotor with the mark facing up. Centrifuge at 9000 rpm (10,000 × *g*) for 35 min at 4 °C. The mark is a reference for the location of the pellet at the end of the centrifugation.
11. Obtain new chilled ultracentrifuge tubes and place them in the laminar flow hood.
12. In the hood, transfer the supernatant from the centrifuged polycarbonate ultracentrifuge tubes to new polycarbonate ultracentrifuge tubes. Do not disturb or collect any of the pellets, as this material consists of cellular debris and LEVs. Weigh and balance the new tubes with PBS. Spin the supernatants in the ultracentrifuge at 30,000 rpm (100,000 × *g*) for 60-80 min (or up to 3 h) at 4 °C. The pellet will be on the side of the tube facing up near the bottom of the tube.
13. At the end of the spin, aspirate as much supernatant as possible without disturbing the pellet and resuspend all pellets in 40-50 mL of PBS. Pellets from the same cell line may be combined in a single tube. Weigh and if needed balance with a centrifuge tube containing PBS.
14. Spin in the ultracentrifuge at 30,000 rpm (100,000 × *g*) for 60-80 min (or up to 3 h) at 4 °C.
15. Carefully aspirate all supernatant, resuspend the pellet in 100 µL of PBS or iodixanol buffer (see Section 4), aliquot and store at -80 °C until further use. Note: when performing immunoblotting analysis, keep in mind that the level of sEV markers and other proteins of interest may vary in different cell lines. The individual user must determine the amount of protein needed to test for the markers.

## 3. Isolation of sEVs from plasma via differential ultracentrifugation

### Materials

1. 1.5-mL Eppendorf tubes.
2. 1× PBS.
3. Plasma.
4. Tabletop Eppendorf centrifuge (5415R).
5. Beckman Coulter 3.5-mL polycarbonate ultracentrifuge tubes (Cat. # 34622).
6. Beckman TLA 100.3 fixed-angle rotor (Cat. # 349490).
7. Beckman L8-70M ultracentrifuge (TLA-100.3 fixed-angle rotor).
8. RIPA lysis buffer: 50 mM Tris, pH 7.5, 150 mM NaCl, 1 mM EDTA, pH 8, 1% NP-40, 0.5% Na-deoxycholate, 0.1% SDS. Add protease inhibitor cocktail just prior to cell lysis (see Section 2).

### Methods

This procedure has been adapted from a previously published protocol^[16]^, and modifications have been added as needed.

#### Pre-clearing steps (plasma)

1. Thaw plasma (3-5 mL) on ice and centrifuge the samples at 1500 × *g* for 10 min at 4 °C.
2. Transfer the supernatant to a new Eppendorf tube, and discard the tube containing the pellet. Note: for each tube, collect the supernatant using a pipette and tilt the tube so that the pellet is on the inside of the tube opposite to the supernatant, making sure not to disturb the pellet.
3. Centrifuge the plasma in the Eppendorf tubes at 12,000 × *g* for 30 min at 4 °C. The plasma may be passed through a 0.22-μm disposable filter at this point or go directly to step 4.
4. Transfer the plasma to 3.5-mL ultracentrifuge tubes. The remaining pellet contains mostly large vesicles which can be resuspended in PBS for storage or they can be lysed in RIPA lysis buffer if further analysis is desired. Note: this protocol is suitable for human plasma or plasma obtained from animal models, in which 3-5 mL are available. For samples of smaller volume, such as from mice, adjust the volume with clean PBS or use smaller ultracentrifuge tubes and rotors (such as the TLA 100.2) as needed. Alternatively, pool samples from multiple mice. As previously described^[17]^, it may also be acceptable to perform EV isolation from mouse plasma using ExoQuick^TM^ or similar PEG precipitation methods^[18]^, followed by iodixanol density gradient separation of EVs from additional debris.

#### Ultracentrifuge steps

1. Centrifuge the plasma from step 4 above at 47,000 rpm (110,000 × *g*) for 2 h at 4 °C using the TLA 100.3 rotor.
2. Wash/resuspend each pellet with 1 mL of PBS.
3. Centrifuge the resuspended pellet at 47,000 rpm (110,000 × *g*) for 2 h at 4 °C.
4. Remove and discard supernatant. The pellet contains predominantly sEVs. Resuspend the pellet in up to 100 µL of PBS for functional analysis or isolation by iodixanol density gradient ultracentrifugation. Alternatively, they can be lysed with RIPA lysis buffer for immunoblotting or iodixanol buffer to separate sEVs using iodixanol gradients (see Section 5). Note: to concentrate the sEV preparation, resuspend and combine pellets from different preparations of the same sample in 100 µL of PBS. To resuspend the pellet, it is advised to cut the pipette tips to avoid lysing the sEVs. If a pellet is particularly difficult to resuspend, brief exposure (1-3 s) to the tabletop mixer can be used as well.
5. Store samples at -80 °C or use for SDS-PAGE immediately.

## 4. Isolation of sEVs from cell culture medium via iodixanol density gradient

### Materials

1. Iodixanol (OptiPrep^TM^, Sigma # 1556) stock solution (60% wt/vol).
2. Buffer solution (0.25 M sucrose, 10 mM Tris, pH 8.0, 1 mM EDTA, pH 7.4).
3. 1× PBS.
4. SETON open-top centrifuge polyallomer tubes.
5. SW55Ti swinging-bucket rotor.
6. Beckman L8-70M Ultracentrifuge.
7. 1.5 mL ultra-microfuge tubes.
8. S55A2 micro-ultracentrifuge rotor.
9. Sorvall^TM^ MTX 150 micro-ultracentrifuge.
10. ABBE-3L refractometer.

### Methods

The sEV pellet isolated from cell lines by differential ultracentrifugation (refer to Section 2) can be further isolated using iodixanol density gradient ultracentrifugation. This protocol has been modified from a previously published protocol^[8]^.

1. In a 3-4 mL ultracentrifuge tube, prepare a discontinuous gradient of 30%, 20%, and 10% iodixanol solutions:
  1. 30% iodixanol (1.6 mL total volume): combine 800 μL of 60% iodixanol/Optiprep^TM^ stock and 800 μL of buffer solution mixed with the sEV pellet;
  2. 20% iodixanol (700 μL total volume): combine 233 μL of 60% iodixanol/Optiprep^TM^ stock with 467 μL of buffer solution;
  3. 10% iodixanol (700 μL total volume): combine 117 μL of 60% iodixanol/Optiprep^TM^ stock with 583 μL buffer.
2. Carefully layer 30%, 20%, and 10% gradient solutions (bottom to top) in SETON tubes.
3. Centrifuge the discontinuous gradient solution at 51,000 rpm (350,000 × *g*) for 70 min at 4 °C in the SW55Ti rotor using the Beckman L8-70M ultracentrifuge.
4. After the spin, collect 10 fractions of 260 μL each in the 1.5-mL ultra-microfuge tubes starting from the top of the tube.
5. In separate Eppendorf tubes, collect 10 μL of each fraction to assess their density with an ABBE-3L refractometer and calculate the density using the conversion table provided by the manufacturer (Optiprep^TM^).
6. Dilute the 250-μL fractions with 1 mL of PBS. Centrifuge at 100,000 × *g* for 70 min at 4 °C in the S55A2 micro-ultracentrifuge rotor.
7. Discard the supernatant and resuspend the pellet with 1 mL of PBS. Centrifuge at 100,000 × *g* for 70 min at 4 °C, in the S55A2 micro-ultracentrifuge rotor to wash the sEVs.
8. Resuspend the resulting pellets for each fraction in 30-80 μL of PBS and store them at -80 °C until further use.

## 5. Isolation of sEVs from plasma via iodixanol density gradient

### Materials

1. Iodixanol (OptiPrep^TM^, Sigma # 1556) stock solution (60% wt/vol).
2. Iodixanol buffer solution (0.25 M sucrose, 10 mM Tris, pH 8.0, 1 mM EDTA, pH 7.4)^[8]^.
3. 1× PBS.
4. Beckman 13-mL ultracentrifuge tubes.
5. Beckman SW55Ti swinging-bucket rotor.
6. Beckman L8-70M Ultracentrifuge.
7. Beckman TLA-100.2 rotor.
8. Beckman Optima^TM^ ultracentrifuge.
9. ABBE-3L refractometer.

### Methods

sEVs can be isolated from patient plasma by differential ultracentrifugation (refer to Section 3) and further isolated using iodixanol density gradient ultracentrifugation. This protocol has been modified from a previously published protocol^[16,19]^.

1. Prepare 40%, 20%, 10% and 5% wt/vol iodixanol solutions by diluting a stock solution (60% wt/vol) of iodixanol (OptiPrep^TM^) with the iodixanol buffer solution.
2. Mix the sEV pellet with stock iodixanol solution to obtain 783 μL of 40% iodixanol-sEV suspension.
3. Next, layer the 783 μL of 20% (wt/vol) iodixanol, 783 μL of 10% (wt/vol) iodixanol, and 652 μL of 5% (wt/vol) on top of the 40% iodixanol-sEV suspension to generate a discontinuous iodixanol gradient.
4. Centrifuge the tubes at 100,000 × *g* for 16 h at 4 °C in the SW55Ti rotor using the Beckman, L8-70M ultracentrifuge.
5. Starting from the top of the tube, collect 10 fractions of 275 μL each.
6. Assess the density of each fraction with the ABBE-3L refractometer.
7. Dilute all fractions and wash them with 1 mL of PBS. Centrifuge the fractions at 100,000 × *g* for 2 h at 4°C, in a TLA-100.2 rotor using an Optima^TM^ TL ultracentrifuge.
8. Resuspend the resulting pellets from each fraction in 30 μL of PBS and store them at -80 °C until further use.

## 6. Isolation of sEVs from tissues via differential ultracentrifugation

The following protocol has been optimized and slightly modified from a previously published protocol^[20]^. It should be noted that a recent manuscript has reported three additional protocols for isolation of large and small EVs from cancer tissue^[21]^.

### Materials

1. EDTA buffer: 10 mM EDTA in PBS (pH 8.2).
2. 1.5-mL Eppendorf tubes.
3. 1× PBS.
4. 2-mL ultracentrifuge Eppendorf tubes.
5. Frozen or fresh tissue.
6. Eppendorf tabletop centrifuge (5415R).
7. S55A2 fixed-angle rotor.
8. SORVALL^TM^ MTX 150 micro-ultracentrifuge.
9. RIPA lysis buffer: 50 mM Tris, pH 7.5, 150 mM NaCl, 1 mM EDTA, pH 8, 1% NP-40, 0.5% Na-deoxycholate, 0.1% SDS. Add protease inhibitor cocktail just prior to cell lysis (see Section 2).

### Methods

1. Set tabletop centrifuge temperature at 4 °C.
2. Cut the tissue into small pieces that can fit into the 1.5-mL Eppendorf tubes.
3. Add 500 μL of EDTA buffer to each tube with tissue pieces.
4. Incubate tissue pieces for 45 min at room temperature.
5. After incubation, centrifuge the sample (EDTA buffer + tissue) at 16,000 × *g* for 20 min at 4 °C using an Eppendorf centrifuge.
6. Transfer the supernatant to 2-mL ultracentrifuge Eppendorf tubes and place the samples in the S55A2 rotor for ultracentrifugation.
7. Centrifuge the samples at 100,000 × *g* for 1 h and 20 min at 4 °C to pellet sEVs in the S55A2 rotor and using SORVALL^TM^ MTX micro-ultracentrifuge.
8. Discard the supernatants if the pellet is visible; if the pellet is not visible, leave around 200 μL at the bottom of the tube.
9. To wash sEVs, add 1 mL of PBS, pipette up and down to resuspend the pellet, and centrifuge the suspension at 100,000 × *g* for 1 h and 20 min at 4 °C in the S55A2 rotor using a SORVALL^TM^ MTX micro-ultracentrifuge.
10. Remove the supernatant and resuspend the pellet in 100 μL of PBS.
11. Store the vesicles at -80 °C or lyse them with RIPA lysis buffer for immunoblotting to test for sEV markers.

## 7. Isolation of LEVs from cell culture medium via differential ultracentrifugation and iodixanol density gradient

### Materials

1. Laminar flow hood.
2. FBS-free cell culture medium.
3. Cell culture medium containing *L*-glutamine and supplemented with 10% FBS and 1% P/S reagent.
4. 1× PBS.
5. Sterile cell culture plates.
6. Sterile 10-mL and 25-mL serological pipettes.
7. 50-mL conical tubes.
8. Tabletop centrifuge with temperature control for 50-mL conical tubes.
9. Beckman polycarbonate ultracentrifuge tubes (65 mL, including the cap assemblies, for use with 45Ti rotor).
10. Beckman 45Ti fixed-angle rotor (Cat. # 339160).
11. Balance.
12. Beckman L8-70M ultracentrifuge (for use with the 45Ti rotor).
13. 1.5-mL Eppendorf tubes.
14. Eppendorf tabletop centrifuge (5415R).

### Methods

1. Culture cells of interest in 20 150-mm plates until they reach 70%-80% confluency.
2. Remove the culture medium and replace it with FBS-free medium; then place the plates in the incubator for 48 h.
3. After 48 h, collect the medium in 50-mL conical tubes in the hood, using a 25-mL pipette. The medium can be combined in tubes from the same cell lines.
4. Set the tabletop centrifuge at 4 °C. Centrifuge the 50-mL conical tubes at 2000 × *g* for 20 min at 4 °C to pellet dead cells and debris.
5. Return to the hood and transfer the supernatant from the 50-mL tubes to the prechilled polycarbonate ultracentrifuge tubes. Each tube can be filled with approximately 65 mL of medium. Note: leave approximately 2-3mL of medium at the bottom to avoid disturbing the pellet.
6. Weigh the polycarbonate ultracentrifuge tubes on the balance. Equalize the weights of tubes for centrifugation by adding sterile PBS to the tubes. Note: ensure that all the rubber stoppers are in place (Beckman 45Ti rotor). Mark one side of each polycarbonate ultracentrifuge tube with a waterproof marker to identify the location of the pellet.
7. Centrifuge the tubes at 9000 rpm (10,000 × *g*) for 35 min at 4 °C in the 45Ti rotor. The pellet obtained consists of LEVs and some cellular debris. Note: in the hood, you can dispose the supernatant or save it for further isolation of sEVs (Section 2). While removing the supernatant, make sure that the pellet is not disturbed. Aspirate as much as possible, leaving no more than 1 mL at the bottom of the tube.
8. Using the 1000-µL pipette, resuspend the pellet in each tube in 1 mL of sterile PBS.
9. Transfer the LEV suspension to 1.5-mL Eppendorf tubes and label them accordingly.
10. Centrifuge the Eppendorf tubes in the tabletop centrifuge at 13,200 rpm (16,000 × *g*) for 40 min at 4 °C.
11. Once the spin is over, return to the hood and aspirate the supernatant without disturbing the pellet.
12. Resuspend the pellet in each tube in 100 µL of sterile PBS. Combine all the Eppendorf tubes in one tube and label accordingly.
13. Centrifuge the sample containing the LEVs at 13,200 rpm (16,000 × *g*) for 40 min at 4 °C.
14. At the end of the spin, carefully aspirate the supernatant, resuspend the LEV pellet in 100 µL of PBS and store at -80 °C for further use.
15. For isolation of LEVs from cell culture medium via iodixanol density gradient ultracentrifugation, please refer to Section 4, where the LEV pellet (described in step 14) will replace the sEV pellet.

## 8. Immunocapture of iodixanol gradient isolated sEVs from cell culture medium or plasma

Immunoisolation of sEVs utilizing antibodies that target sEV markers (CD9, CD63 and CD81) or other transmembrane proteins expressed on sEVs, e.g., prostate-specific membrane antigen, offers several advantages. Examples of such advantages are: (1) isolation of concentrated sEV sample; (2) removal of contaminants other than sEVs which may obscure the results; and (3) comparison of potential subpopulations of sEVs. Materials and methods utilized for the immuno-capture of sEVs^[16]^ are as follows.

### Materials

1. Cell culture- or plasma-derived iodixanol gradient-isolated sEVs (Section 4, 5).
2. Monoclonal antibodies to tetraspanins (CD9, CD63 and CD81) or to cancer-specific markers present on sEVs (e.g., prostate-specific membrane antigen). Note: use antibodies that specifically target an epitope in the extracellular domains of target proteins and are suitable for immunoprecipitation applications.
3. Isotype immunoglobulins.
4. Dynabeads^TM^ M-270 epoxy beads (Invitrogen, Cat. # 14301).
5. Microbalance.
6. 1.5-mL Eppendorf tubes.
7. Buffers (Dynabeads M-270 epoxy beads, Invitrogen, Cat. # 14301): Buffer A: 0.1 M sodium phosphate buffer (pH 7.4) [dissolve 2.62 g NaH_2_PO_4_·H_2_O (MW 137.99) and 14.42 g Na_2_HPO_4_·2H_2_O (MW 177.99) in distilled water, adjust pH if necessary and adjust volume to 1 L]; Buffer B: 3 M ammonium sulfate (stock solution): dissolve 39.6 g (NH_4_)_2_SO_4_ (MW 132.1) in 0.1 M sodium phosphate buffer (pH 7.4) and adjust volume to 100 mL; Buffer C: 0.1 M citrate, pH 3.1: dissolve 2.10 g citric acid (C_6_H_8_O_7_·H_2_O, MW 210.14) in 90 mL of distilled water, adjust to pH 3.1 and adjust volume to 100 mL.
8. 1× PBS.
9. Vortex.
10. DynaMag^TM^ magnet (Invitrogen).
11. Incubator (37 °C) with tilt rotation.
12. Mini Mixer (4 °C).
13. RIPA Lysis buffer: 50 mM Tris, pH 7.5, 150 mM NaCl, 1 mM EDTA, pH 8, 1% NP-40, 0.5% Na-deoxycholate, 0.1% SDS. Add protease inhibitor cocktail just prior to cell lysis (see Section 2).

### Methods

The following protocol for immunocapture of sEVs has been optimized and slightly modified from Dynabeads^TM^ M-270 epoxy beads (Invitrogen, Cat. # 14301).

#### Resuspend beads

1. Weigh out 5 mg of lyophilized superparamagnetic Dynabeads (~3.3 × 10^8^ beads) on the microbalance. Put beads in 1.5-mL Eppendorf tubes and resuspend in 1 mL of Buffer A.
2. Mix well by vortexing for 30 s followed by 10 min incubation at room temperature with tilting and rotating.
3. Place the Eppendorf tube in the DynaMag^TM^ magnet for 1 min and discard the supernatant.
4. Remove the tube from the magnet and again resuspend the washed beads in 1 mL of Buffer A; mix well by vortexing for 30 s.
5. Place the tube on the DynaMag^TM^ magnet for 1 min and discard the supernatant.

#### Coupling antibodies to the beads

1. Resuspend the washed beads from above in the same volume of Buffer A as calculated for the antibody volume and vortex the suspension. For example, for 5 mg of beads, ~100 µg of antibody is needed. Accordingly, if antibody concentration is 1 mg/mL, a 100-µL antibody volume is needed. Thus, 100 µL of Buffer A is needed for the resuspension of beads.
2. Add ~100 µL of antibody as in the example above and thoroughly vortex the suspension.
3. Add the same volume of Buffer B (100 µL as in the example above). Note: with 5 mg of beads in a total of 300 µL of buffer-antibody mix, the coupling concentration is at ~1.1 × 10^9^ beads/mL.
4. Incubate bead-antibody-buffer mix from step 3 for 16-24 h at 37 °C with slow tilt rotation. Note: beads should not settle during the incubation period.
5. After the incubation, place the Eppendorf tubes on the DynaMag^TM^ magnet for 2 min, and then remove the supernatant. Note: ensure the collection of any beads adhering to the cap.
6. Wash the antibody-coated beads four times with 1 mL of PBS, each time resuspending the beads in PBS and applying them to the DynaMag^TM^ magnet for 2 min during each wash.
7. Resuspend the antibody-coated beads to the desired concentration in PBS (e.g., a volume of 165 µL gives a bead concentration of ~2 × 10^9^ beads/mL).

#### Immunocapture and Elution of sEVs

1. Add iodixanol gradient-purified sEVs (30-40 µg) to 165 µL of antibody-coupled beads mentioned above.
2. Incubate overnight with tilting and rotating at 4 °C on a mini mixer to capture the target protein-specific sEVs.
3. Place the tube on the DynaMag^TM^ magnet for 4 min to collect the bead-sEV complexes at the tube wall. Pipet off the supernatant.
4. Wash the bead-sEV complexes 3 times using 1 mL of PBS each time by resuspending the beads and applying the DynaMag^TM^ magnet for 2 min during each wash.
5. The immunocaptured sEVs can either be lysed with RIPA lysis buffer to proceed to immunoblotting analysis or eluted in the following steps.
6. For elution of sEVs from bead-antibody complex, add 10-100 µL of Buffer C to the beads with immobilized sEVs and mix well by tilting and rotating for 2 min.
7. Place the Eppendorf tubes on the DynaMag^TM^ magnet for 2 min and transfer the supernatant containing the target protein-specific purified sEVs to a clean tube. Note: change Eppendorf tubes before elution to avoid eluting off non-specific contaminants binding to the tube walls.

## 9. Comments on limitations of most frequently used isolation techniques

### Differential ultracentrifugation

The isolation of EVs via differential ultracentrifugation has advantages for users since this technique is cost-effective, yields a large amount of EVs and is relatively easy to learn and perform. Differential ultracentrifugation protocols are well established and readily available to users. This method also has some disadvantages that users need to keep in mind. When isolating EVs through differential ultracentrifugation, co-precipitation of protein aggregates, cellular debris, small non-EV structures and EV-associated RNA can occur; this can lead to decreased sample purity. It should also be noted that the yield of EVs varies depending on the conditions and the cell lines used^[5,22-25]^.

### Density gradient

Density gradient isolation allows to preserve the EVs in the gradient by making iso-osmotic solutions at all densities. However, this method may result in low yield of small vesicles and sample loss. Disadvantages associated with density gradient are that larger vesicles can be harder to separate due to their similar sedimentation rates which lead to some inaccuracy in the sample analysis. For functional assays, if using sucrose, it is advised to remove the density gradient medium since it may interfere with some functional assays^[5,25-27]^.

### Immunocapture

Immunocapture is a very specific technique used to isolate a targeted set of vesicles that are homogeneous and contain similar protein content. This method works efficiently even if the starting material is small. Unfortunately, this technique is not cost-effective and not suited for processing large sample volumes. In addition, bound EVs cannot be used for functional assays since samples will contain antibodies that can affect EV-cell interactions; also eluting the EVs from the beads may not be possible. To isolate the desired population of EVs, it is important to have specific EV markers for each class of EVs^[5,14,23-25,27,28]^.

## 10. Additional methods

Additional isolation methods are summarized below although not in detail.

### Size-exclusion chromatography

Size-exclusion chromatography (SEC) is now widely used for EV separation. Whether it is through high performance liquid chromatography or gravity-based chromatography at the benchtop, the main principle is as follows. The sample is passed through a column with porous beads made of various materials (predominantly Sepharose). The pore size needs to be small to avoid trapping of the EVs, which will therefore elute from the column sooner than small contaminating proteins such as albumin, which can co-precipitate in other common EV isolation methods such as ultracentrifugation. SEC is also useful for cleaning up antibody or membrane dye-stained EVs, as the labeled sEVs will elute sooner than the unbound dye, qdot, and so on^[29,30]^. Most protocols for SEC-based purification of EVs still involve differential ultracentrifugation or other pre-clearing steps using high performance liquid chromatography in which the sample is pumped through the column and the fractions collected at pre-set intervals based on either a set amount of time or volume. Previous work has shown that the retention time for the EVs is approximately 7.5 min^[29,31]^. However, this may vary according to flowrate through the column and pore size of the column material, so it is recommended to collect and examine multiple fractions for the presence of EVs when working with new, previously untested samples and conditions.

### Tangential flow filtration

Tangential flow filtration (TFF) is a relatively new EV isolation technique known to be able to rapidly process large volumes of cell culture medium or other fluids. It is a fast, efficient and reliable method for isolation of biologically active EVs^[32,33]^. Although there are several methods for EV isolation and purification, there is a need for the development of methods for large-scale clinical grade EVs. TFF is being recognized as a promising method for isolation of EVs and development of cGMP compliant EV purification protocols^[32-34]^.

Unlike the traditional dead-end filtration method, where the sample flows perpendicular to the membrane and eventually clogs the membrane pores, in the case of TFF, the fluid flows tangentially across the surface of a semi-permeable membrane with a particular molecular weight cut-off. This causes less clogging and buildup on the membrane^[34,35]^ and therefore better removal of contaminants. It is important to choose the correct molecular weight cut-off membrane to make sure that EVs are retained and do not pass through the membrane. The transmembrane pressure allows the supernatant sample to flow across the membrane, allowing the separation of EVs and non-EV components. EVs are retained (retentate) and circulated back to the feed reservoir while the contaminants pass through the membrane (filtrate). TFF is also used for concentration and diafiltration for buffer exchange and salt removal. Therefore, at the end of a TFF run, a high concentration of purified EVs is obtained^[34,35]^. A number of studies in the field have shown the isolation of highly concentrated and bioactive EVs and have indicated that TFF is an efficient method for obtaining EVs^[32,33]^.

## 11. Conclusion

We describe here the methods that are most commonly used to isolate sEVs from tissues and biological fluids; we also describe the methods that are most commonly used to isolate sEVs and LEVs from cell culture medium. We apologize for not being able to describe other methods for EV isolation or commercially available kits due to space constraints. With a need for transparency and consistency in the field of EV research, investigators are encouraged to report the chosen methods used for isolation and characterization, such as expression of sEV and LEV markers, nanoparticle tracking analysis^[36,37]^, tunable resistive pulse sensing, flow cytometry, laser tweezers Raman spectroscopy, digital ELISA, digital PCR and transmission electron microscopy^[17,38,39]^.

## Declarations

### Copyright

© The Author(s) 2020.
